# The reporting quality of acupuncture-related traumatic adverse events: a systematic review of case studies in Korea

**DOI:** 10.1186/s12906-024-04421-5

**Published:** 2024-03-13

**Authors:** Sung-A Kim, Ji-Su Lee, Tae-Hun Kim, Seunghoon Lee, Jae-Dong Lee, Jung Won Kang

**Affiliations:** 1 Department of Clinical Korean Medicine, Graduate School, Kyung Hee University, Seoul, South Korea; 2 Department of Acupuncture and Moxibustion, Kyung Hee University Medical Center, Seoul, South Korea; 3 Korean Medicine Clinical Trial Center, Korean Medicine Hospital, Kyung Hee University, Seoul, South Korea; 4 Department of Acupuncture & Moxibustion, College of Korean Medicine, Kyung Hee University, 23 Kyungheedae-ro, Dongdaemun-gu, Seoul, 02447 South Korea

**Keywords:** Acupuncture, Adverse events, Case reports, Systematic review, Trauma injury

## Abstract

**Background:**

Acupuncture is known for a harmless treatment when administered by well-trained clinicians. However, multiple case reports of traumatic adverse events (AEs) related to acupuncture treatments continue to be published in literature. In this review, we evaluated the reporting quality and conducted causality assessments of case studies that have reported acupuncture-related traumatic AEs in Korea.

**Methods:**

Eight databases were searched from their inception to January 2024. Only Korean case studies that reported traumatic AEs following acupuncture procedures were included without any language restrictions. Reporting quality was evaluated based on patient characteristics, AEs, and acupuncture practice. Causality was assessed using the modified WHO-UMC causality criteria.

**Results:**

Twenty-eight studies were included from a total of 1,154 identified studies. The quality of reporting in the included studies was low overall. While the descriptions of patient characteristics and AEs were relatively well detailed, most information on acupuncture practice was not reported at all. During the causality assessment, only three (10.7%) studies were judged to be “certain”. Twelve (42.9%) studies were “unassessable” because they inadequately described the information necessary for decision-making. It was practically difficult to establish the causality between acupuncture and AEs, as well as the appropriateness of acupuncture practice.

**Conclusions:**

Insufficient and inappropriate reporting was observed in most case studies reporting acupuncture-related traumatic AEs in Korea. To overcome these limitations, we have suggested tentative guidelines in the form of a set of items that should be reported by future authors who plan to publish case studies on acupuncture-related traumatic AEs in a clinical setting.

**Supplementary Information:**

The online version contains supplementary material available at 10.1186/s12906-024-04421-5.

## Background

Acupuncture is a safe procedure when performed by qualified practitioners. However, multiple case reports of minor or serious adverse events (AEs) related to acupuncture treatments continue to be published in literature. The AEs of acupuncture can be classified into four major groups: (1) trauma to organs or tissues, (2) infection, (3) local AEs, and (4) miscellaneous AEs such as dizziness or syncope [1]. Among these, traumatic acupuncture-related AEs are induced by piercing any organ or tissue in the human body using acupuncture needles during or after acupuncture. Well-known traumatic AEs cover pneumothorax, cardiac tamponade, hemopericardium, hematoma, neuropathy, pseudoaneurysm, and the migration of broken needles [2]. These AEs are clinically critical because they can cause serious harm to the patient’s health and necessitate the quick delivery of first aid and subsequent definitive management [3].

Although case studies reporting these AEs are considered to have a low level of evidence, case studies can be a valuable source for clinicians and researchers to identify the characteristics of AEs. They can reflect real-world clinical practice rather than controlled clinical settings in compliance with previously established treatment protocols in clinical trials. In addition, case reports can describe specific features, such as patient characteristics, details of therapy, and other susceptibility factors involved in AEs [4]. Using rigorously documented case studies, clinicians can determine the seriousness, preventability, and causality of AEs, which could further help in determining the safety of acupuncture [5].

Previous systematic reviews (SRs) have reported that the overall quality of case studies reporting AEs after acupuncture treatment is too low to confirm a causal relationship between acupuncture and AEs [6, 7]. Unless case reports rigorously follow a proper reporting standard, they would not only be insufficient for data analysis but also fail to suggest reliable evidence for clinical practice. However, in the field of acupuncture-related AEs, there are no established reporting guidelines for case studies, such as the CAse REport (CARE) guidelines [8]. Additionally, causality assessment tools such as the WHO-Uppsala Monitoring Center (WHO-UMC) criteria and the Naranjo algorithm, which can ensure the reliability and validity of reporting AEs, are still limited to only adverse drug events [9, 10].

This study aimed to preliminarily evaluate the current status of case studies focused on acupuncture-related traumatic AEs in Korea. In this review, we first assessed the quality of reporting and performed a causality appraisal. Furthermore, considering the characteristics of acupuncture procedures and traumatic AEs, we have suggested essential items for case studies to improve their reporting quality and to develop practical reporting guidelines.

## Methods

This was a systematic review of case reports (or series) on traumatic acupuncture-related AEs. In this methodological review, we assessed the reporting quality of the included case reports; therefore, it was thought that protocol registration was not required. The study was conducted according to the Preferred Reporting Items for Systematic reviews and Meta-Analyses literature search extension (PRISMA-S) [11]. The PRISMA-S checklist was presented in Supplement 1.

### Literature searches

Case reports (or series) published in Korea were retrieved from the following eight databases since inception to 16 January 2024: MEDLINE via PubMed, EMBASE via Ovid, the Cochrane Central Register of Controlled Trials (CENTRAL), Database Periodical Information Academic (DBPIA), Korean Medical Database (KMBASE), Korean Studies Information Service System (KISS), National Discovery for Science Leaders (NDSL), and Oriental Medicine Advanced Searching Integrated System (OASIS). In addition, we manually searched the reference lists of published studies and websites to identify relevant articles that could not be retrieved through our electronic search. Our search terms were “traumatic adverse event” and “acupuncture” according to the language of each database. The search strategy was modified based on the characteristics and structures of individual databases. Detailed search strategies for each database are provided in Supplement 2.

### Data selection

Only case reports (or series) published in Korea were included without language restrictions. Randomized controlled trials, nonrandomized controlled trials, and qualitative and prospective studies were excluded. Studies were eligible if they reported traumatic AEs of acupuncture that ranged from injury to internal organs (i.e., pneumothorax and cardiac tamponade), central or peripheral nerves, and blood vessels (i.e., hemorrhage, blood vessel rupture, or pseudoaneurysm) to migration of broken acupuncture needles. In this review, we only included manual acupuncture. Other acupuncture-related treatment modalities, such as electroacupuncture, pharmacoacupuncture, thread-embedding therapy, gold needle, warm acupuncture, intramuscular stimulation, and unidentified fragments presumed to be acupuncture, were excluded, as these could induce other causative processes. Titles and abstracts of the identified case reports (or series) were screened by two independent authors (S-AK and J-SL). The full texts of all eligible articles were obtained and selected according to the inclusion criteria. Any differences of opinion between the two researchers were resolved by a third author (JWK).

### Data extraction

Data were independently extracted by two authors (S-AK and J-SL) using predefined data extraction forms. The following data were extracted: the first author’s name, year of publication, number of cases, patient information, details of acupuncture treatment, classification of traumatic AEs according to system organ class (SOC) of the MedDRA hierarchy, and author’s conclusion with quotations. Disagreements between the reviewers were resolved by a third arbitrator (JWK).

### Assessment of reporting quality of information related to the patients, adverse events and acupuncture practice

As no assessment tool is available for these types of reporting quality evaluations, the quality assessment criteria were established based on previous studies and group discussions [6]. 

Data were divided into three main categories to assess the reporting quality of the included studies. The first category was a description of patient characteristics, including age, sex, reason for acupuncture treatment, and patient history. The second was the information on AEs that covered the duration from the last acupuncture treatment and onset of traumatic symptoms, radiologic or pathologic findings, final diagnosis, follow-up clinical outcome, the association between needling site and affected lesion, and consideration of other risk factors for AEs. The third category included the details of the acupuncture treatment, which comprise the type of practitioner and acupuncture, selected acupuncture points, depth of insertion, stimulation method, patient posture during treatment, and acupuncture retention time. Data were rated as “well demonstrated (WD),” “demonstrated but not sufficient for judgement (DS),” “not demonstrated (ND)” and “not applicable (NA)” [6]. We judged the data as WD when adequate information was fully described and as ND when the information was not described at all. A rating of DS was given when the information was present but was not sufficiently descriptive. If the data could not be reported because of the specificity of the study, it was evaluated as NA.

Two authors individually assessed and discussed each item (S-AK and J-SL). Disagreements between the two authors were resolved by a third arbitrator (JWK).

### Causality assessment

Causality between acupuncture and AEs was assessed using the modified WHO-UMC causality assessment criteria: (1) “Certain” if there was definitive diagnostic test with plausible time relation and the AE could not be explained by other risk factors, (2) “Probable” if the events were unlikely to be attributed to other reasons and there was reasonable time relationship to acupuncture treatment, (3) “Possible” if the AE could also be explained by other factors but there was reasonable time relation to acupuncture treatment, (4) “Unlikely” if there were other plausible reasons and a time relationship to acupuncture treatment that makes a relationship improbable (but not impossible), (5) “Conditional/Unclassified” if additional data for judgment was needed for assessment, and (6) “Unassessable/Unclassifiable” if we could not evaluate causality due to insufficient or contradictory information or if the study only suggested relationship between acupuncture and events without verifiable evidence [12].

We also appraised the appropriateness of acupuncture treatment to evaluate whether the practice could be a potential cause of the AEs: (1) “Appropriate” if none of the acupuncture procedures could be a probable cause for the events, (2) “Inappropriate” if any improperly performed process of acupuncture practice might be the probable cause for the events, and (3) “Unclear” if we could not determine causality due to the lack of detailed description of the acupuncture treatment [6].

## Results

### Search results

**Fig. 1:**
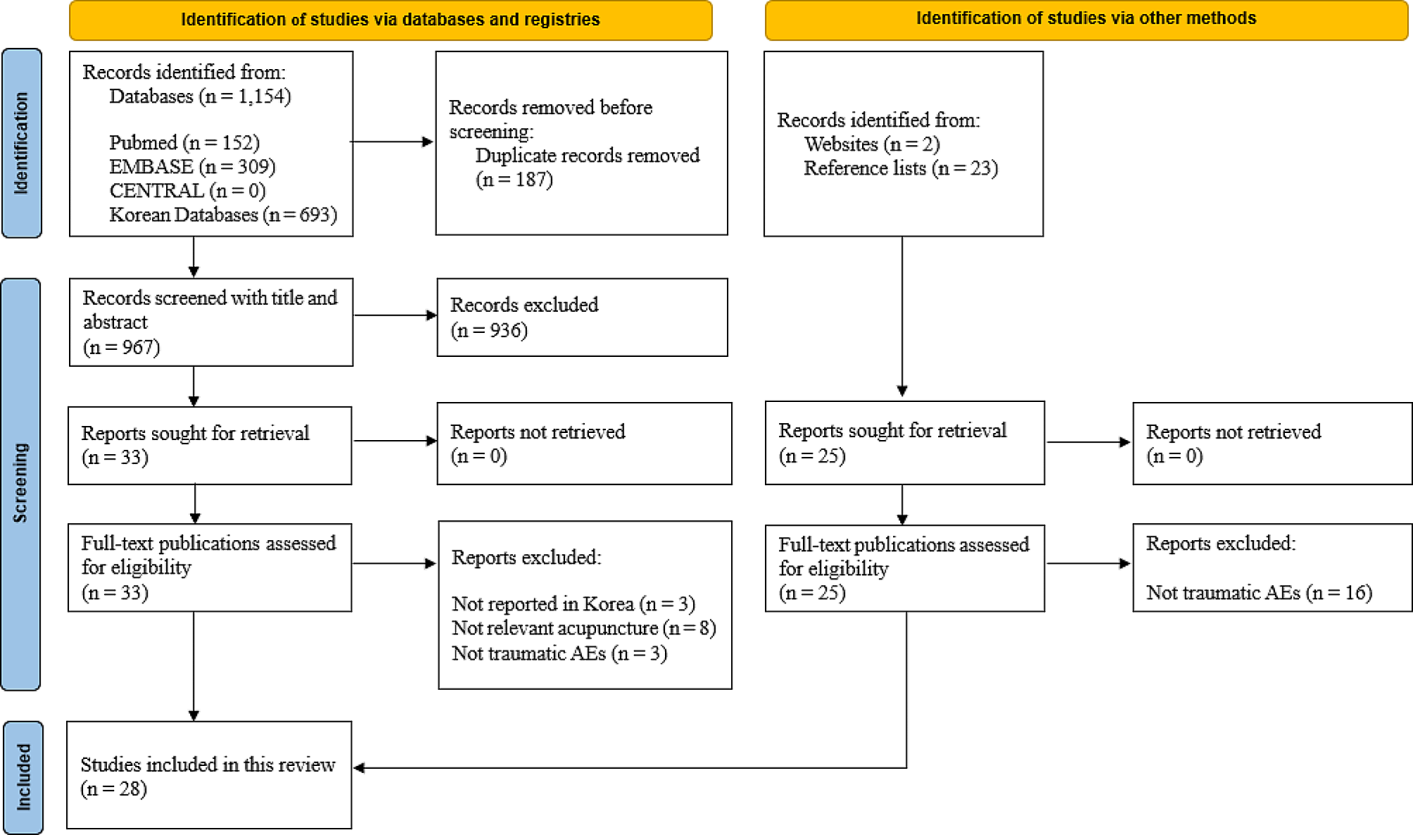
Study flow chart. *AEs, adverse events

A total of 1,154 records were identified from the electronic databases; 25 additional records were identified through other sources. After eliminating duplicate studies, 992 studies were filtered by screening their titles and abstracts. Fifty-eight full-text articles were assessed for eligibility for several reasons. Of these, 28 were included in this review. These search procedures have been presented in the PRISMA 2020 flowchart demonstrated in Fig. 1.

### Study characteristics

The studies included twenty-six single case reports and two case series. Both of the case series included two independent cases of AE, one of which was an AE directly related to acupuncture treatment and the other to non-acupuncture treatments, such as intramuscular stimulation and surgery.

AEs were classified according to SOCs, which were also used in the Common Terminology Criteria for Adverse Events (CTCAE) version 5.0. to group the disease or symptoms according to etiology and manifestation site. In this review, the most commonly damaged organ in AEs was the nervous system. The traumatic AEs included nervous system trauma in eight studies, vascular trauma in five, gastrointestinal trauma in four, cardiac trauma in four, eye trauma in three, respiratory in two, renal and urinary trauma in two studies.

The AEs included the following specific disorders; nervous system trauma (e.g. cerebrospinal fluid rhinorrhea, central nervous system injury, and sensory neuropathy in median nerve), vascular trauma (e.g. pseudoaneurysm, hematoma, hemorrhage from the inferior epigastric artery, and ileocecal vein rupture), gastrointestinal trauma (e.g. cecal hemorrhage, pneumoretroperitoneum, retropharyngeal hematoma, and retroperitoneal hematoma), cardiac trauma (e.g. cardiac tamponade and hemopericardium), respiratory trauma (e.g. pneumothorax), renal and urinary trauma (e.g. vesicocutaneous fistula, and perirenal hematoma), and eye trauma (e.g. ocular perforation) (Table 1).

**Table 1:** Patient characteristics

Study ID (author, year)	Patient’s age (gender)	SOC classification	Final diagnosis (case numbers)
Bahn (1999) [34]	45 (M)	Nervous system	Cerebrospinal fluid rhinorrhea (1)
Chun (2014) [19]	48 (F)	Cardiac	Cardiac tamponade (1)
Chung (2008) [16]	66 (M)	Vascular	Limb hematoma (1)
Chung (2016) [40]	62 (M)	Eye	Ocular perforation (1)
Chung (2020) [33]	55 (M)	Gastrointestinal	Iatrogenic retroperitoneal hematoma (1)
Ha (2009) [17]	36 (M)	Gastrointestinal	Retropharyngeal hematoma (1)
Her (2013) [14]	62 (M)	Cardiac	Cardiac tamponade (1)
Hong (2006) [24]	46 (M)	Vascular	Pseudoaneurysm (1)
Hwang (2008) [26]	25 (F)	Gastrointestinal	Pneumoretroperitoneum (1)
Jeon (2004) [22]	56 (F)	Nervous system	Cisterna magna infarction (1)
Kang (2013) [30]	42 (F)	Eye	Ocular perforation (1)
Koo (2022) [21]	64 (F)	Vascular	Vertebro-vertebral arteriovenous fistula (1)
Kim (2006) [25]	66 (M)	Nervous system	Spinal cord infarction (1)
Kim (2009) [27]	71 (F)	Renal and urinary	Perirenal hematoma (1)
Kim (2011a) [36]	78 (M)	Cardiac	Cardiac tamponade (1)
Kim (2011b) [18]	55 (M)	Nervous system	Compression neuropathy of superficial peroneal nerve and deep peroneal nerve (1)
Kim (2011c) [37]	54 (F)	Cardiac	Hemopericardium (1)
Lee (2001) [13]	27 (F)	Respiratory	Pneumothorax (1)
Lee (2005) [23]	75 (F)	Gastrointestinal	Cecal hemorrhage (1)
Lee (2008) [35]	47 (M)	Nervous system	Median sensory neuropathy (1)
Lee (2020) [39]	58 (F)	Eye	Ocular perforation (1)
Moon (2013a) [29]	71 (F)	Vascular	Hemorrhage from the inferior epigastric artery (1)
Moon (2013b) [38]	55 (M)	Vascular	Ileocecal vein rupture (1)
Park (2013) [28]	69 (M)	Nervous system	Acute spinal subdural hematoma (1)
Park (2019) [31]	47 (M)	Nervous system	Spinal subdural hematoma (1)
Sung (2016) [15]	32 (M)	Respiratory	Pneumothorax (1)
Woo (2020) [32]	66 (F)	Nervous system	Cervical spinal cord injury (1)
Yeo (2021) [20]	54 (F)	Renal and urinary	Vesicocutaneous fistula (1)

*SOC, system organ class

The causality between AEs and acupuncture was not assessed in any of the included studies.

### Reporting quality assessment

#### The reporting quality of information regarding the patient characteristics and acupuncture-related traumatic adverse events

Patient information, including age and sex (0%, hereinafter referred to as the percentage of studies with insufficiently or not reported information), reasons for seeking acupuncture treatment (25%), and medical history (35.7%), were described in most studies.

Information regarding AEs, including the duration from the last acupuncture treatment and onset of AEs symptoms (32.1%), diagnostic test (0%), and clinical outcome (0%), was generally well documented in most studies. However, information required to judge causality, including an explanation of the association between needling site and affected lesion (71.4%) and consideration of other risk factors for AEs (100%), was either not documented at all or insufficiently documented in most studies (Table 2).

**Table 2:** Reporting quality assessment of information regarding patients and adverse events

	Patient’s information			AEs information				
Study ID (author, year)	Age (gender)	Reason for AT	Patient medical history	Duration from last AT and onset of symptom	Diagnostic test	Clinical outcome (follow up)	Explanation on the association between needling site and lesion	Consideration for other risk factors for AEs
Bahn (1999) [34]	45 (M)	WD	WD	WD	WD	WD	WD	ND
Chun (2014) [19]	48 (F)	ND	DS	WD	WD	WD	WD	ND
Chung (2008) [16]	66 (M)	WD	DS	WD	WD	WD	DS	ND
Chung (2016) [40]	62 (M)	WD	DS	WD	WD	WD	DS	ND
Chung (2020) [33]	55 (M)	WD	WD	WD	WD	WD	ND	ND
Ha (2009) [17]	36 (M)	ND	WD	WD	WD	WD	DS	ND
Her (2013) [14]	62 (M)	WD	WD	WD	WD	WD	WD	ND
Hong (2006) [24]	46 (M)	ND	WD	ND	WD	WD	ND	ND
Hwang (2008) [26]	25 (F)	WD	ND	DS	WD	WD	ND	ND
Jeon (2004) [22]	56 (F)	WD	WD	WD	WD	WD	DS	ND
Kang (2013) [30]	42 (F)	WD	WD	ND	WD	WD	DS	ND
Koo (2022) [21]	64 (F)	WD	DS	WD	WD	WD	DS	ND
Kim (2006) [25]	66 (M)	WD	WD	DS	WD	WD	DS	DS
Kim (2009) [27]	71 (F)	WD	DS	WD	WD	WD	ND	ND
Kim (2011a) [36]	78 (M)	WD	WD	WD	WD	WD	WD	ND
Kim (2011b) [18]	55 (M)	WD	WD	WD	WD	WD	DS	ND
Kim (2011c) [37]	54 (F)	WD	ND	WD	WD	WD	DS	ND
Lee (2001) [13]	27 (F)	WD	WD	WD	WD	WD	WD	ND
Lee (2005) [23]	75 (F)	WD	WD	DS	WD	WD	DS	ND
Lee (2008) [35]	47 (M)	WD	ND	DS	WD	WD	WD	ND
Lee (2020) [39]	58 (F)	WD	WD	WD	WD	WD	WD	DS
Moon (2013a) [29]	71 (F)	WD	WD	WD	WD	WD	DS	ND
Moon (2013b) [38]	55 (M)	ND	WD	DS	WD	WD	DS	ND
Park (2013) [28]	69 (M)	DS	DS	WD	WD	WD	DS	ND
Park (2019) [31]	47 (M)	WD	WD	WD	WD	WD	DS	ND
Sung (2016) [15]	32 (M)	WD	WD	DS	WD	WD	WD	ND
Woo (2020) [32]	66 (F)	ND	ND	WD	WD	WD	DS	ND
Yeo (2021) [20]	54 (F)	ND	WD	DS	WD	WD	DS	ND

*AT, acupuncture treatment; AEs, adverse events; DS, demonstrated but not sufficient for judgment; NA, not applicable; ND, not demonstrated; WD, well demonstrated

#### The reporting quality of information regarding the acupuncture practice

All the AEs were reported to occur after the acupuncture procedure in the included studies. However, the information on acupuncture practice, which is essential to determine whether the traumatic AEs were actually caused by acupuncture itself, was either not described at all or described insufficiently in most studies. The reporting status of individual items related to the acupuncture practice was as follows: type of practitioner (hereinafter referred to as percentage of studies with insufficiently or not reported information, 39.3%), needle type (85.7%), names of points or locations (71.4%), depth of insertion (92.9%), direction of insertion (92.9%), needle stimulation method (92.9%), posture during treatment (92.9%), and needle retention time (100%) (Table 3).

**Table 3:** Reporting quality assessment of the information regarding acupuncture practice

	Details of acupuncture practice								
Study ID (author, year)		Practitioner’s type	Needle type (diameter, length)	Names of points or locations	Depth of insertion	Direction of insertion	Needle stimulation method	Posture during treatment	Needle retention time
Bahn (1999) [34]		WD	WD	WD	ND	ND	ND	ND	ND
Chun (2014) [19]		WD	ND	WD	ND	ND	ND	ND	ND
Chung (2008) [16]		ND	ND	DS	ND	ND	ND	ND	ND
Chung (2016) [40]		ND	ND	DS	ND	ND	ND	ND	ND
Chung (2020) [33]		WD	DS	ND	ND	ND	ND	ND	ND
Ha (2009) [17]		WD	DS	DS	ND	ND	ND	DS	ND
Her (2013) [14]		ND	WD	WD	ND	ND	ND	ND	ND
Hong (2006) [24]		ND	ND	ND	ND	ND	ND	ND	ND
Hwang (2008) [26]		WD	ND	ND	ND	ND	ND	ND	ND
Jeon (2004) [22]		ND	ND	DS	ND	ND	ND	ND	ND
Kang (2013) [30]		WD	ND	DS	ND	ND	ND	ND	ND
Koo (2022) [21]		ND	ND	DS	ND	ND	ND	ND	ND
Kim (2006) [25]		WD	ND	DS	ND	ND	ND	ND	ND
Kim (2009) [27]		WD	ND	ND	ND	ND	ND	ND	ND
Kim (2011a) [36]		WD	ND	WD	ND	ND	ND	ND	ND
Kim (2011b) [18]		WD	ND	DS	ND	ND	ND	ND	ND
Kim (2011c) [37]		WD	ND	DS	ND	ND	ND	ND	ND
Lee (2001) [13]		WD	WD	WD	WD	DS	WD	ND	ND
Lee (2005) [23]		WD	ND	DS	ND	ND	ND	ND	ND
Lee (2008) [35]		WD	ND	WD	ND	ND	ND	ND	ND
Lee (2020) [39]		WD	ND	WD	ND	WD	ND	ND	ND
Moon (2013a) [29]		DS	ND	DS	ND	ND	ND	ND	ND
Moon (2013b) [38]		ND	DS	DS	ND	ND	ND	ND	ND
Park (2013) [28]		WD	ND	DS	ND	ND	ND	ND	ND
Park (2019) [31]		DS	DS	DS	ND	ND	ND	ND	ND
Sung (2016) [15]		WD	WD	WD	WD	WD	WD	WD	ND
Woo (2020) [32]		ND	ND	DS	ND	ND	ND	WD	ND
Yeo (2021) [20]		ND	ND	DS	ND	ND	ND	ND	ND

*AT, acupuncture treatment; DS, demonstrated but not sufficient for judgment; NA, not applicable; ND, not demonstrated; WD, well demonstrated

### Causality assessment

#### Causality assessment according to the modified WHO-UMC criteria

All the included studies concluded with a phrase that implicitly asserted a strong association between acupuncture treatment and AEs. However, causality assessment using the modified WHO-UMC criteria revealed that 42.9% of the studies were unassessable or unclassifiable because of poor reporting. Other studies were evaluated as certain (10.7%), probable or likely (10.7%), possible (14.3%), unlikely (21.4%), or conditional or unclassified (0%).

The three cases assessed as certain included a case of cardiac tamponade in a patient who had to undergo surgery because of the insertion of an acupuncture needle (6 cm long) into the pericardium, and two cases of pneumothorax. One of the patients with pneumothorax complained of dyspnea right after the treatment (depth of acupuncture needle insertion: 1 to 3 cm into the trapezius and rhomboid muscles), and the other complained of dyspnea during the treatment of the subscapularis muscle [13–15]. Based on whether a reasonable time relationship was established, we evaluated these three cases as probable and another four cases as possible. Six cases were evaluated as unlikely because the temporal relationship was inappropriate in these studies, and the possibility of other factors was highly suspected [16–21]. Other risk factors included the presence of an underlying disease, and patient’s medical and social history. For example, a patient with a vesicocutaneous fistula with a history of radiotherapy at the site of needling, or a patient with a cardiac tamponade detected after cardiopulmonary resuscitation with a history of chemotherapy of the breast. Twelve cases were evaluated as unassessable or unclassifiable as it was not possible to judge the causal relationship because of a lack of patient information, AEs or acupuncture procedure [22–33].

Even with the uncertain causality between acupuncture treatment and AEs, the authors of these studies concluded by using misleading expressions regarding the causality between acupuncture and AEs (Table 4).

**Table 4:** Causality assessment based on the modified WHO-UMC criteria

Study ID (author, year)	Authors’ conclusion on the causal relationship between acupuncture and adverse events (Quotation from studies)	Causality assessment*
Bahn (1999) [34]	“We experienced an interesting case of traumatic CSF rhinorrhea associated with acupuncture.”	Probable/likely
Chun (2014) [19]	“The diagnosis of cardiac tamponade induced by an acupuncture needle should be considered when unexplained shock after an acupuncture procedure at the chest wall is found.”	Unlikely
Chung (2008) [16]	“We present the case of a 66-year-old male with a long-standing history of type 2 diabetes who presented with a painful left lower extremity after needle acupuncture and was diagnosed with acute compartment syndrome.”	Unlikely
Chung (2016) [40]	“We observed a case of ocular perforation and endophthalmitis following ocular acupuncture treatment. This case illustrates the dangers of intraocular acupuncture therapy.”	Possible
Chung (2020) [33]	“In conclusion, we report a case of reversible duodenal obstruction due to a large retroperitoneal hematoma after acupuncture therapy.”	Unassessable/Unclassifiable
Ha (2009) [17]	“We report here on a case of a retropharyngeal hematoma following acupuncture therapy and we review the relevant literature.”	Unlikely
Her (2013) [14]	“We report a case of 62-year-old man with cardiac tamponade due to coronary artery injury after acupuncture into the substernum.”	Certain
Hong (2006) [24]	“We had experienced … pseudoaneurysms that occurred after acupuncture … and the patients were treated successfully with interventional and surgical procedures.”	Unassessable/Unclassifiable
Hwang (2008) [26]	“This report describes a case of pneumoretroperitoneum that developed after acupuncture.”	Unassessable/Unclassifiable
Jeon (2004) [22]	“It is probable that the acupuncture needle had been inserted deep enough to enter the cisterna magna and provoked a small hemorrhage in the cistern.”	Unassessable/Unclassifiable
Kang (2013) [30]	“We report the case of ocular perforation and retinal tear with normal retinal function and persistent visual field impairment due to retinal nerve fiber severance, caused by acupuncture treatment, which has not yet been reported in Korea.”	Unassessable/Unclassifiable
Koo (2022) [21]	“We herein report a case of a traumatic vertebro-vertebral arteriovenous fistula that occurred following the application of oriental acupuncture in the posterior neck region.”	Unlikely
Kim (2006) [25]	“This report describes a case of spinal cord infarction after acupuncture.”	Unassessable/Unclassifiable
Kim (2009) [27]	“Recently, we experienced … perirenal hematoma in patients without blood coagulation abnormalities (one case induced by acupuncture in oriental medicine clinic…).”	Unassessable/Unclassifiable
Kim (2011a) [36]	“In the following case, cardiac tamponade was caused by epigastric acupuncture.”	Probable/likely
Kim (2011b) [18]	“The purpose of this report is to describe what we believe to be the first case of delayed superficial and deep peroneal nerve compressive neuropathy caused by fibrotic mass formed by neglected broken acupuncture needle.”	Unlikely
Kim (2011c) [37]	“We report a case of hemopericardium that occurred shortly after acupuncture … “	Possible
Lee (2001) [13]	“In order to make known that acupuncture can cause pneumothorax in reality and to prevent more acupuncture-related adverse effect cases in future, this report was made.”	Certain
Lee (2005) [23]	“We report the case of 75-year-old woman with an intramural hematoma who took cumadin after acupuncture …”	Unassessable/Unclassifiable
Lee (2008) [35]	“This case highlights the possibility of a focal peripheral nerve lesion resulting from acupuncture, possibly caused by herbal medicine coating the needle.”	Possible
Lee (2020) [39]	“Authors present a case of a patient with vitreous hemorrhage and ocular perforations caused by periocular acupuncture therapy in both eye, …”	Probable/likely
Moon (2013a) [29]	“We report a rare case of haemorrhage from the inferior epigastric artery, which was injured after acupuncture.”	Unassessable/Unclassifiable
Moon (2013b) [38]	“We report here on a case of an iliocecal vein rupture following acupuncture therapy and we review the relevant literature.”	Possible
Park (2013) [28]	“In this report, we present an atypical case of SDH with unilateral weakness after acupuncture …”	Unassessable/Unclassifiable
Park (2019) [31]	“This patient has functional dysfunction due to SSDH after the acupuncture, which shows that even severe complications may occur after acupuncture.”	Unassessable/Unclassifiable
Sung (2016) [15]	“For acupuncture treatment to the interscapular area, the depth and manual technique should be carefully performed.”	Certain
Woo (2020) [32]	“The authors report a case of cervical neurological damage that occurred after an acupuncture procedure.”	Unassessable/Unclassifiable
Yeo (2021) [20]	“To the best of our knowledge, this is the first case to report the occurrence of bladder injury with VCF following acupuncture. “	Unlikely

*Causality was assessed according to the WHO-UMC criteria based on the information provided within the reports on AEs

#### Appropriateness of acupuncture treatment

Due to incompletely described information, the appropriateness of acupuncture practice could not be appraised in 22 studies (78.6%). The other six studies were assessed as inappropriate (21.4%) because of the unauthorized practitioner’s treatment or the unusual depth (or direction) of needle insertion considering the anatomical location (Table 5).

**Table 5:** Appraisal of the appropriateness of acupuncture treatment

Study ID (author, year)	Appropriateness of acupuncture treatment*	
Bahn (1999) [34]	Inappropriate	
Chun (2014) [19]	Unclear	
Chung (2008) [16]	Unclear	
Chung (2016) [40]	Unclear	
Chung (2020) [33]	Unclear	
Ha (2009) [17]	Unclear	
Her (2013) [14]	Inappropriate	
Hong (2006) [24]	Unclear	
Hwang (2008) [26]	Unclear	
Jeon (2004) [22]	Unclear	
Kang (2013) [30]	Inappropriate	
Koo (2022) [21]	Unclear	
Kim (2006) [25]	Unclear	
Kim (2009) [27]	Unclear	
Kim (2011) [36]	Unclear	
Kim (2011) [18]	Unclear	
Kim (2011) [37]	Unclear	
Lee (2001) [13]	Unclear	
Lee (2005) [23]	Unclear	
Lee (2008) [35]	Unclear	
Lee (2020) [39]	Inappropriate	
Moon (2013a) [29]	Unclear	
Moon (2013b) [38]	Inappropriate	
Park (2013) [28]	Unclear	
Park (2019) [31]	Unclear	
Sung (2016) [15]	Inappropriate	
Woo (2020) [32]	Unclear	
Yeo (2021) [20]	Unclear	

*Appropriate, if none of the acupuncture procedure could be a probable cause for the events; Inappropriate, if any process of acupuncture practice might be the probable cause for the events; Unclear, if we could not determine the causality due to the lack of detailed description of the acupuncture treatment

## Discussion

This review evaluated the quality of case studies reporting acupuncture-related traumatic AEs in Korea. In the included twenty-eight case studies, no fatal AEs were reported. However, the reporting quality of these studies was generally low. Therefore, it was difficult to confirm whether there was a clear causal relationship between acupuncture and traumatic AEs. Most studies tended to meticulously document the diagnosis, radiologic or pathologic findings, and clinical outcomes of AEs; however, the information related to acupuncture practice and possible alternative etiologies was not fully documented. Hence, causality was rated as unlikely or unassessable for most of the included studies.

In this review, we identified several specific features. First, the reporting quality of the included studies varied significantly according to the qualifications of the authors. Most information stated in the two case studies reported by Korean Medical doctors who performed the treatment procedure or observed the AEs was appraised as well described. These authors presented all the necessary details, sufficiently analyzed the possibility of other alternative factors, and also specified inappropriate procedural aspects that could be related to AE in discussion Sects. [13, 15]. In contrast, the reporting quality of twenty-six studies, which were reported by surgeons or physicians handling AEs in the clinic, was mostly rated as not demonstrated or insufficiently demonstrated, except for diagnostic tests and clinical outcomes done by themselves [14, 16–40]. Neither information on acupuncture practice nor consideration for other risk factors of AEs was considered in these studies. Furthermore, the information gathering was not complete as it was based on merely querying the patient in a cursory manner. This might reflect the unique clinical context in Korea, in which the licenses of Korean Medical doctors and conventional medical doctors are legally separated. As acupuncture practice can be implemented only by institutionally qualified Korean Medical doctors, it would be hard for Western medical doctors to provide a detailed description of the acupuncture treatment process according to STandards for Reporting Interventions in Clinical Trials of Acupuncture (STRICTA) [41]. In addition, acupuncture practice consists of multiple components; therefore, its complexity would pose a challenge for doctors not trained in the procedures. They would not be precisely aware of the particular aspects of acupuncture practice that are associated with traumatic AEs [42]. It is doubtful whether other risk factors of AEs were considered in these studies. When examining the relevance of acupuncture-related traumatic AEs, it is fundamental to impartially weigh the possibility of both acupuncture and other risk factors, such as the presence of an underlying disease, trauma history, or spontaneous occurrence. This negatively affects reporting objectivity and quality.

Second, causality in 64.3% of the included studies was assessed as unlikely or unassessable using the WHO-UMC criteria. However, most of the authors of the included studies hastily concluded that acupuncture resulted in AEs only because of the temporal relationship, without explaining the association between the needling site and the lesion. Whether acupuncture needles penetrated injured tissues is a crucial factor in traumatic AEs. Therefore, it should be discussed whether the lesion and needled region are consistent using an anatomical approach. For this, it could be the most objective evidence to clarify the distance and direction between the lesion and the puncture site using radiological findings [43]. In addition, information regarding the needle stimulation methods (e.g., needle rotation, lift thrust, or respiration) and the patient’s posture during the treatment time should be included. Without rigorous investigation of these critical factors, there is a danger of overinterpretation and information bias that could further lead to misunderstanding the post hoc fallacy as causality [44]. For example, there was a case report of a pneumothorax that had been mistaken as an acupuncture-related traumatic AE only due to the temporal sequence. However, it was determined to be spontaneous after close examination of the whole acupuncture procedure by Korean Medical doctors; they could not observe any trauma or needle fragments on chest CT [45].

Third, the appropriateness of the acupuncture practice in all included studies was rated as unlikely or inappropriate. In order to discuss the appropriateness of acupuncture practice, it is necessary to distinguish whether AEs occurred even though the standardized procedure was properly performed by a licensed and well-trained practitioner. Competent practitioners have proven that acupuncture is safe [46, 47]. Furthermore, especially in Korea, the safety of acupuncture performed by a practitioner is institutionally guaranteed as they are granted a license by the Ministry of Health and Welfare after finishing study at a college of Korean Medicine for six years and passing the national examination. Nevertheless, five cases were rated as inappropriate acupuncture practice. Malpractice types were unlicensed practitioners or inappropriate needle type, depth, direction of needle insertion that should not have been chosen [14, 15, 30, 34, 38, 39]. For example, according to the authorized acupuncture textbook, acupuncture points located at the anterior thoracic region, inferior to the xiphisternal junction, and upper abdomen should not be penetrated deeply or upward, and acupuncture points at the trapezius should be penetrated after holding up the muscle, not toward the thorax, and at a depth of 10–20 mm, in consideration of the personal anatomical distance to the lungs [2]. This implies that these AEs were all caused by malpractice rather than acupuncture treatment itself. Hence, these AEs would have been avoidable if the procedure had been performed precisely according to standard principles.

This study had some limitations. First, we only assessed case studies reported in Korea; therefore, the results are not representative of AEs occurring in other countries. In Korea, the safety of acupuncture is institutionally guaranteed by granting a license to Korean Medical doctors. However, in other countries, acupuncture can be practiced not only by doctors but also by acupuncturists, nurses, and physiotherapists with varying levels of training [48]. Second, we systematically reviewed only case studies (or series). Although case studies have the advantage of descriptively stating unusual AEs in real-world practice, there could be a possibility of exaggerating the harmful effects of acupuncture. The risk of bias, such as measurement and recall bias, is inherent in these studies because they are anecdotal, heterogeneous, and retrospective in nature. Furthermore, it is not possible to estimate the incidence rate, which should have been assessed for AEs, because case studies are retrospective. Third, the search for relevant studies was limited. Most studies were not relevantly indexed, such as those indexed to specific diagnosed diseases rather than trauma or adverse events. Although we might have missed poorly or inaccurately indexed articles, we tried to avoid overlooking the relevant studies as much as possible by additionally implementing manual searching.

Based on the current status identified in this review, we formed a provisional guideline for authors who are considering the submission of case studies on traumatic AEs related to acupuncture (Table 6). Compared with a previously suggested reporting guideline for acupuncture-related AEs, it includes more detailed information regarding traumatic features, such as the location, depth, and direction of acupuncture needle insertion, posture shift during treatment, or individual vulnerability to bleeding tendency. Furthermore, it classifies the information into two categories, as the authors of the included studies were usually Western Medical doctors unfamiliar with the acupuncture regimen: (1) “Required”: that should be reported as a minimum, confirmed from the patient and practitioners, (2) “Desirable”: that should preferably be written in detail, especially reflecting the characteristics of traumatic AEs following acupuncture procedure. The required information includes essential items such as patient age, sex, social history, underlying diseases with duration, consideration of the possibility of spontaneous occurrence and other traumatic history, duration between last acupuncture treatment and onset of AE symptoms, radiologic or pathologic findings, clinical diagnosis and medical management, causality assessment using the WHO-UMC criteria, certification of practitioner with affiliation (local, hospital, or unauthorized office), acupuncture needle type with length, anatomical location of needling site, depth and direction of acupuncture insertion, posture during treatment, and number of treatment sessions. Desirable information included the height, weight, body mass index (BMI), dosage of medication and other treatments (anticoagulant, radiotherapy), individual vulnerability (sternal foramen, weight loss, cardiomegaly, chronic obstructive pulmonary disease, or vascular malformation), specific timeline of last acupuncture treatment, onset of AE symptoms, diagnosis of AE, radiological evidence indicating distance between needling site and trauma lesion, clinical outcome with follow-up (after a few weeks or years, whether there is recurrence or not), points or lesions using WHO standard acupuncture point locations, appraisal for the appropriateness of acupuncture, clinical training and experience period of practitioner, needle diameter, name of acupuncture points with numbers of needles, stimulation method (rotation, lift-thrust, or respiration), posture shift during treatment and concurrent treatment (chuna manual therapy, spinal manipulation, electroacupuncture, or pharmaco-acupuncture).

**Table 6:** Recommendation items for case reports on acupuncture-related traumatic adverse events

	Required	Desirable
Characteristic of patients		
Demographic data	Age, sex, social history (alcohol, smoking, job, etc.)	Height, Weight, or Body Mass Index (BMI)
Medical information	Underlying diseases with duration	Dosage of medication and other treatments (anticoagulation, radiotherapy, etc.)
Consideration for individual risk factors for AEs	Consideration on possibility of spontaneous occurrence and other traumatic history	Individual vulnerability (sternal foramen, weight loss, cardiomegaly, chronic obstructive pulmonary disease, vascular malformation, etc.)
Description regarding adverse events		
Time relation between last acupuncture treatment and onset of symptom	Duration between last acupuncture treatment and onset of AE symptom	Specific timeline of last acupuncture treatment, onset of AE symptom, and diagnosis of AE
Radiologic or pathologic findings	Radiologic or pathologic findings	Radiological evidence indicating distance between needling site and trauma lesion
Clinical outcomes	Clinical diagnosis and medical management	Clinical outcome with follow-up (after a few weeks or years, whether there is recurrence or not)
Explanation of the association between needling site and lesion	Anatomical location	Points using WHO standard acupuncture point locations
Causality	Causality assessment using WHO-UMC criteria	Appraisal of the appropriateness of acupuncture
Description of acupuncture practice		
Type of practitioner	Certification of practitioner with affiliation (local, hospital, unauthorized office)	Clinical training and experience period
Acupuncture type	Needle length	Needle diameter
Acupuncture points	Anatomical location	Name of acupuncture points with numbers of needles
Acupuncture stimulation	Depth and direction of insertion	Stimulation method (rotation, lift-thrust, respiration)
Posture of patient	Posture during treatment	Posture shift during treatment
Treatment regimen	Number of sessions	Concurrent treatment (chuna manual therapy, spinal manipulation, electroacupuncture, pharmaco-acupuncture, etc.)
Others		
Title	Include acupuncture type (manual acupuncture, electroacupuncture, auricular acupuncture, etc.) and AEs	Include type of adverse events such as trauma or injury
Author	Consult Korean Medicine doctor for the procedure of acupuncture treatment	Include qualified Korean Medicine doctor as authors
Discussion	Discussion of previous reports of adverse events, disease specific characteristics, and other patient related risk factors	Based on appraisal of causality assessment and appropriateness of acupuncture in neutral attitude

* AE, adverse event; WHO, World Health Organization

† Desirable, information that would be recommended to be written in detail, especially reflecting the characteristic of traumatic AEs following acupuncture; Required, information that should be reported as a minimum

Future case studies should comply with these specifically tailored recommendations as well-conducted case studies could provide guidance for safe treatment practices (i.e., malpractice type and clinical manifestations of patients), educational implications in identifying unique, unusual, or serious AEs, and balanced evidence for medical disputes. Nevertheless, if methodological rigor is not ensured, indiscreetly or misreported case studies may distort the fact that acupuncture is safe when performed by qualified practitioners.

## Conclusion

We assessed the quality of reporting and conducted a causality appraisal of case studies on acupuncture-related traumatic AEs in Korea. The reported quality of the included case studies was low. The causal relationship between acupuncture and traumatic AEs could not be properly identified because of a lack of crucial information. To ensure better practice of reporting traumatic AEs of acupuncture in case reports, we have suggested tentative guidelines in the form of a set of items that should be reported by future authors who plan to publish case studies on acupuncture-related traumatic AEs. We expect that this will contribute to the improvement of the reporting quality of case studies and will be a valuable step toward future consensus-based reporting guidelines. However, further research and the development of standardized guidelines for acupuncture-related traumatic AEs are required.

### Electronic supplementary material

Below is the link to the electronic supplementary material.

Supplementary Material 1

Supplementary Material 2

## Data Availability

The data that support the findings of this study are available within the article and supplementary material.

## Abbreviations

AEs: Adverse events
AT: Acupuncture treatment
DS: Demonstrated but not sufficient for judgment
NA: Not applicable
ND: Not demonstrated
SOC: System organ class
SR: Systematic review
WD: Well demonstrated
WHO: World Health Organization
WHO-UMC: World Health Organization -Uppsala Monitoring Center
