# Supplementary material for: The reporting quality of acupuncture-related traumatic adverse events: a systematic review of case studies in Korea
Source: BMC Complement Med Ther. 2024 Mar 13;24:120. doi: 10.1186/s12906-024-04421-5 (PMC10935981; doi:10.1186/s12906-024-04421-5)
Supplement: Supplementary file 2 — Supplementary Material 2 [file 12906_2024_4421_MOESM2_ESM.docx]

Appendix. Supplemenary Material

Supplement. 1. Search strategy

A1. CENTRAL

1. 'adverse event' OR 'adverse effect' OR 'adverse reaction' OR 'adverse outcome' OR 'side effect' OR 'undesirable event' OR 'undesirable effect' OR 'undesirable reaction' OR ‘undesirable outcome' OR 'complication'

2. 'acupuncture' OR 'acupuncture therapy' OR 'point, acupuncture' OR ‘Dry needling’

3. 'injury' OR 'injuries' OR 'injuries’ OR 'injury' OR 'trauma' OR 'traumatic injury' OR 'traumatic lesion' OR 'wounds’ OR ‘pneumothorax’ OR ‘cardiac tamponade’ OR ‘Hemopericardium’ OR ‘hemorrhage’ OR ‘hematoma’ OR ‘neuropathy’ OR ‘pseudoaneurysm’ OR ‘rupture’ OR ‘broken needle’

4. 'case report' OR ‘case series’ OR ‘case series’

5. #1 and #2 and #3 and #4

A2. EMBASE

1. acupuncture/exp

2. acupuncture needle/exp

3. acupuncture therapy/exp

4. dry needling/exp

5. acupuncture points/exp

6. acupuctur*.mp.

7. MTrPs.mp.

8. or/1-7

9. ((adverse OR side OR undesirable) NEXT/2 (effect* OR reaction* OR event* OR outcome*)).ti,ab.

10. complication.ti,ab.

11. or/9-10

12. 'injury'/exp OR 'injuries' OR 'injuries’ OR 'injury' OR 'trauma' OR 'traumatic injury' OR 'traumatic lesion' OR 'wounds’

13. ‘pneumothorax’/exp OR ‘cardiac tamponade’/exp OR ‘Hemopericardium’/exp OR ‘hemorrhage’/exp OR ‘hematoma’/exp OR ‘neuropathy’/exp OR ‘pseudoaneurysm’/exp OR ‘rupture’/exp OR ‘broken needle’/exp

14. or/12-13

15. ((case or cases) NEXT/2 (report or reports or series or study or studies)).tw.

16. 8 and 11 and 14 and 15

A3. MEDLINE

1. “case reports"[Publication Type]

2. "case reports"[Title/Abstract]

3. “case series"[Title/Abstract]

4. “case study"[Title/Abstract]

5. #1 OR #2 OR #3 OR #4

6. ("adverse"[All Fields] OR "adversely"[All Fields] OR "adverses"[All Fields]) AND ("event"[All Fields] OR "event s"[All Fields] OR "events"[All Fields])

7. "complicances"[All Fields] OR "complicate"[All Fields] OR "complicated"[All Fields] OR "complicates"[All Fields] OR "complicating"[All Fields] OR "complication"[All Fields] OR "complication's"[All Fields] OR "complications"[Subheading] OR "complications"[All Fields]

8. #6 OR #7

9. "acupuncture"[MeSH Terms]

10. "acupuncture"[Title/Abstract]

11. "acupuncture therapy"[Title/Abstract]

12. "acupuncture points"[Title/Abstract]

13. "dry needling"[MeSH Terms]

14. "dry needling"[Title/Abstract]

15. #9 OR #10 OR #11 OR #12 OR #13 OR #14

16. "injurie"[All Fields] OR "injuried"[All Fields] OR "injuries"[Subheading] OR "injuries"[All Fields] OR "wounds and injuries"[MeSH Terms] OR ("wounds"[All Fields] AND "injuries"[All Fields]) OR "wounds and injuries"[All Fields] OR "injurious"[All Fields] OR "injury's"[All Fields] OR "injuryed"[All Fields] OR "injurys"[All Fields] OR "injury"[All Fields]

17. "trauma"[All Fields]

18. "pneumothorax" OR "cardiac tamponade" OR "Hemopericardium" OR "hemorrhage" OR "hematoma" OR "neuropathy" OR "pseudoaneurysm" OR "rupture" OR "broken needle“

19. #16 OR #17 OR #18
